# Prevalence of allergen sensitization, most important allergens and factors associated with atopy in children

**DOI:** 10.1590/1516-3180.2013.1315502

**Published:** 2013-10-01

**Authors:** Raquel Prudente de Carvalho Baldaçara, Maria de Fátima Marcelos Fernandes, Leonardo Baldaçara, Wilson Tartuce Aun, João Ferreira de Mello, Mario Cesar Pires

**Affiliations:** I MD, MSc. Assistant Professor in the Department of Medicine, Universidade Federal do Tocantins (UFT), Palmas, Tocantins, Brazil.; II MD, MSc. Head of the Diagnostic and Therapeutic Division, Department of Allergy and Immunology, Hospital do Servidor Público Estadual de São Paulo (HSPE), São Paulo, Brazil.; III MD, PhD. Associate Professor in the Department of Medicine, Universidade Federal do Tocantins (UFT), Palmas, Tocantins, Brazil.; IV MD. Head of the Immunology Section, Department of Allergy and Immunology, Hospital do Servidor Público Estadual de São Paulo (HSPE), São Paulo, Brazil.; V MD, PhD. Director of the Department of Allergy and Immunology, Hospital do Servidor Público Estadual de São Paulo (HSPE), São Paulo, Brazil.; VI MD, PhD. Head of the Diagnostic and Therapeutic Division, Department of Dermatology, Hospital do Servidor Público Estadual de São Paulo (HSPE), São Paulo, Brazil.

**Keywords:** Hypersensitivity, Child, Risk factors, Allergy and immunology, Immunologic techniques, Hipersensibilidade, Criança, Fatores de risco, Alergia e imunologia, Técnicas imunológicas

## Abstract

**CONTEXT AND OBJECTIVE:**

Knowledge of the profile of allergen sensitization among children is important for planning preventive measures. The objective of this study was to assess the prevalence and profile of sensitization to inhaled allergens and food among children and adolescents in an outpatient population in the city of Palmas.

**DESIGN AND SETTING:**

Cross-sectional study at outpatient clinics in Palmas, Tocantins, Brazil.

**METHODS:**

Ninety-four patients aged 1-15 years who were attending two pediatric outpatient clinics were selected between September and November 2008. All of the subjects underwent clinical interviews and skin prick tests.

**RESULTS:**

A positive skin prick test was observed in 76.6% of the participants (72.3% for inhalants and 28.9% for food allergens). The most frequent allergens were *Dermatophagoides pteronyssinus* (34%), cat epithelium (28.7%), dog epithelium (21.3%), *Dermatophagoides farinae* (19.1%), *Blomia tropicalis* (18.1%), cow's milk (9.6%) and grasses (9.6%). A positive skin prick test correlated with a history of atopic disease (odds ratio, OR = 5.833; P = 0.002), a family history of atopic disease (OR = 8.400; P < 0.001), maternal asthma (OR = 8.077; P = 0.048), pet exposure (OR = 3.600; P = 0.012) and cesarean delivery (OR = 3.367; P = 0.019).

**CONCLUSION:**

*Dermatophagoides pteronyssinus* was the most frequent aeroallergen and cow’s milk was the most prevalent food allergen. There was a positive correlation between a positive skin prick test and several factors, such as a family history of atopic disease, maternal asthma, pet exposure and cesarean delivery

## Abstract (pt)

**CONTEXTO E OBJETIVO:**

O conhecimento sobre o perfil da sensibilização a alérgenos em crianças é importante para o planejamento de medidas preventivas. O objetivo deste estudo foi avaliar a prevalência e perfil de sensibilização a alérgenos inalados e alimentares em crianças e adolescentes em uma população ambulatorial na cidade de Palmas.

**TIPO DE ESTUDO E LOCAL:**

Estudo transversal em unidades ambulatoriais em Palmas, Tocantins, Brasil.

**MÉTODOS:**

Foram selecionados 94 pacientes com idades entre 1 a 15 anos em 2 ambulatórios de pediatria entre setembro e novembro de 2008. Todos os sujeitos foram submetidos a entrevistas clínicas e testes cutâneos de puntura.

**RESULTADOS:**

Um teste cutâneo de puntura positivo foi observado em 76,6% dos participantes (72,3% para inalantes, 28,9% para alérgenos alimentares). Os alérgenos mais frequentes foram Dermatophagoides pteronyssinus (34%), epitélio de gato (28,7%), epitélio de cão (21,3%), *Dermatophagoides farinae* (19,1%), *Blomia tropicalis* (18,1%), leite de vaca (9,6%) e gramíneas (9,6%). Um teste cutâneo de puntura positivo foi relacionado à história de doença atópica (razão de chances RC = 5,833, P = 0,002), história familiar de atopia (RC = 8,400, P < 0,001), asma materna (RC = 8,077, P = 0,048), exposição a animal de estimação (RC = 3,600, P = 0,012) e parto cesáreo (RC = 3,367, P = 0,019).

**CONCLUSÃO:**

*Dermatophagoides pteronyssinus* foi o aeroalérgeno mais prevalente e, dentre alérgenos alimentares, o leite de vaca. Houve correlação positiva entre o teste cutâneo e alguns fatores, como história familiar de atopia, asma materna, exposição a animais domésticos e parto cesáreo.

## INTRODUCTION

The World Allergy Organization defines atopy as "the personal or familial tendency to produce immunoglobulin E (IgE) antibodies in response to low allergen doses and to develop typical conditions, such as asthma, rhinitis or eczema".^1-3^ This definition describes immunoreactivity but does not include the presence of clinical symptoms. Atopy is the most important risk factor for development of allergic diseases; however, an atopic patient may yield a positive test in response to an allergen without developing symptoms.^2,3^

This phenomenon may be due to the existence of immunity (natural tolerance) or a suboptimal level of IgE antibodies that is insufficient to produce symptoms.^2,3^ Therefore, patients can be asymptomatic and continue to yield positive allergen test results, which would indicate development of immunity (acquired tolerance) against the allergen. Both situations represent atopy without allergy.

In the industrialized world, it is estimated that 30-50%^2,4^ of the pediatric population presents allergen sensitization. However, many patients will never exhibit allergic symptoms,^4^ which indicates that atopy is not the only factor that is responsible for the development of these allergic disorders. In Brazil, the International Study of Asthma and Allergies in Childhood (ISAAC) study found that the prevalences of asthma, rhinitis and atopic eczema were 21%, 39% and 8%, respectively.^5^ Phase 3 of the ISAAC study found that the highest rates of asthma and eczema were in the northern region (asthma 19.9%, rhinitis 32% and eczema 11.1%).^6^

Over the past decades, there have been parallel increases in the frequencies of atopic diseases and allergen sensitization, which suggests that these increases are related. Because important variations in population genetics could not have occurred over such a short period of time, the underlying causes may correspond to changes in environmental factors.^2^ Two of these factors are food allergen exposure during the first two years of life and cesarean delivery, which are associated with the manifestations of asthma, infant wheezing and allergic rhinitis.^7,8^ During the first months of childhood, allergen sensitizations to cow's milk and eggs occur predominantly.^9^ Thereafter, children may become sensitive to inhaled allergens and may develop allergic respiratory diseases. Early exposure to dust mites during childhood poses a risk of development of asthma.^10^ Among the air allergens, dust mites (*Dermatophagoides pteronyssinus, Dermatophagoides farinae *and *Blomia tropicalis*) are the main source of allergens that are involved in the pathophysiology of allergic respiratory diseases.^11-13^

Investigations of atopic disease may be performed on inhalants and food extracts *in vitro* using the specific IgE test and *in vivo* using the skin prick test. Both methods enable determination of type I hypersensitivity and assist in diagnosing the phenotypicmanifestations of atopic diseases.^1^ However, the skin prick test is more sensitive than the specific IgE test.^14^ Infants born to atopic parents with percutaneous sensitization to aeroallergens are at increased risk of aeroallergen sensitization during infancy, which can persist up to the age of two years.

Personal quality of life is affected by asthma, rhinitis and atopic eczema, and these diseases have an economic impact in Brazil due to their high prevalences.^11^ The capitals of the northern region, such as Belém and Manaus, have higher prevalences of asthma and eczema than other regions in Brazil.^6^ Early diagnosis of atopic diseases makes it possible to begin preventive measures, which reduces the clinical expression of allergic diseases. Therefore, understanding the prevalence of allergen sensitization among patients in various cities is important for implementing preventive measures. Among such cities, Palmas is located in the Amazon region between the Amazon forest and the Cerrado (a vast tropical savanna in Brazil) and is an area of increasing population size. 

### OBJECTIVE

In this study, we aimed to describe the prevalence of allergen sensitization and the distribution of the most important allergens among children aged 1-15 years who were attending two pediatric outpatient clinics in the city of Palmas, Tocantins, Brazil. In addition, we investigated the factors that were associated with the presence of atopy among these patients. 

### METHODS 

### Participants 

We selected a total of 99 patients who were 1-15 years of age and who were attending two pediatric outpatient clinics at primary care level in Palmas, a relatively large city (population around 228,332)^15^ in the state of Tocantins, northern Brazil, between September and November 2008. These children were taken to the outpatient clinic to be monitored in relation to their growth and development, and the clinics emphasized preventive healthcare. 

The inclusion criteria were that the subjects should be children and adolescents who were 1-15 years of age and who were assessed at two pediatric outpatient clinics in the city of Palmas, state of Tocantins. Patients were excluded from the study if they had been hospitalized; had used systemic corticoids or other immunosuppressive medications within 30 days of the start of the study; had used anti-histamine therapy within ten days of the skin prick test; presented severe clinical disease such as cardiopathy, neuropathy, nephropathy or neoplasia; presented fever or extensive dermatological lesions; or did not complete all parts of the study; or additionally, if the guardians failed to provide informed consent. 

All of the children who were enrolled in the study were evaluated by a family doctor at the outpatient clinic. After the patients and their caregivers agreed to participate in the study, the children were sequentially assessed by the researchers, who included a pediatrician and an allergist. 

The sample size was calculated for α = 0.05 and β - 1 = 80%, and a sensitization rate of 30% in the sample studied was expected (as demonstrated in previous studies in the community). A minimum number of 92 patients was found to be needed for the study, based on calculations using the Fleiss methodology.

### Clinical history and examination

The patients were assessed for symptoms that were suggestive of allergic disease, based on their clinical history, physical examinations and the ISAAC (International Study of Asthma and Allergies in Childhood) questionnaire.^5^ The ISAAC study maximized the value of epidemiological research on asthma, allergic rhinoconjunctivitis and atopic eczema through facilitation from international collaboration.^5^

The possible risk factors for atopic diseases that could be correlated with a positive skin prick test were as follows: family history of atopic disease (including maternal asthma), cesarean delivery, passive smoking, intrauterine tobacco exposure, artificial feeding before six months of life, premature birth and recurrent respiratory infections (more than six respiratory infections per year or more than three lower respiratory tract infections per year).^16^

### Skin prick test (SPT)

The skin prick test (SPT) extracts were standardized in International Units (IU) for the following three categories of allergens: inhaled allergens, including dust mites (*Dermatophagoides pteronyssinus*, *Dermatophagoides farinae* and *Blomia tropicalis*), cockroaches (*Periplaneta americana* and *Blattella germanica*), pollens (*Lolium perenne* and *Dactillus glomerata*) and grass mixes (*Dactillus glomerata*, *Festuca pratensis*, *Pheleum pratense*, *Poa pratensis* and *Lolium perenne*); food allergens, including cow's milk, casein, beta-lactoglobulin, soybean, egg white, egg yolk, fish, cacao, corn, peanut, shrimp and wheat; and other allergens, including cat epithelium, dog epithelium, latex, and *Candida albicans*. A histamine concentration of 10 mg/ml was used as the positive control, and a saline solution was used as the negative control (provided by the IPI-ASAC-Brazil laboratory). A positive SPT was defined as a wheal diameter ≥ 3 mm larger than that of the saline control after 15 minutes^11^ and a positive reaction to at least one of the allergens.^17^ The wheal size was measured using the formula: (*D+d*)/2, where D was the maximum diameter and d was the perpendicular diameter.^17^

### Data analysis

The data were analyzed using the Statistical Package for the Social Sciences (SPSS), version 13.0. The differences between the mean values of the subjects who were positive and negative for skin prick tests and the patients with and without atopic disease were tested using non-parametric analyses. The prevalence rates were calculated for the entire group and the subgroups, and the differences were tested using chi-square analysis. The odds ratios (ORs) and their 95% confidence intervals (CIs) were calculated for each risk factor according to each case definition. Logistic regression analysis was used to adjust for potential confounding variables and included significant variables (P < 0.10). Non-significant variables were detected by backwards elimination using the Wald statistic.

### Ethical issues 

This study was approved by the Ethics Committee of the Institute for Medical Care for State Public Servants (Instituto de Assistência Médica ao Servidor Público Estadual, IAMSPE), São Paulo, Brazil (no. 065/07) and by the Municipal Health Department of Palmas (Secretaria de Saúde Municipal de Palmas, Semus), Palmas, Tocantins, Brazil. The guardians of all the participants provided written informed consent before their entry into the study. Participation in the study was voluntary.

### RESULTS 

Out of the 99 patients who were examined, 94 were available to finish the study and provided complete data. Of these 94 patients, 60 (63.8%) were male and 34 (36.2%) were female. The average age was 5.34 years (standard deviation, SD: ± 3.582) with a median age of 5.00 years and a mode of 4.00 years. Seventy-two participants (76.6%) had positive SPTs, 68 (72.3%) were positive for aeroallergens and 27 (28.9%) were positive for food allergens (Table 1). The most frequent allergens that resulted in positive SPTs were *Dermatophagoides pteronyssinus* (34%), cats (28.7%), dogs (21.3%), *Dermatophagoides farinae* (19.1%), *Blomia tropicalis* (18.1%), cow's milk (9.6%) and grass (9.6%).

Personal histories of atopic disease were obtained from 75 subjects (79.8%): 63.8% of these patients reported asthma, 70.2% reported rhinitis and 50.0% reported atopic eczema (Table 1).

**Table 1:** Frequency of prick test positivity for atopic diseases among 1 to 15-year-old children and adolescents in the city of Palmas, Tocantins, Brazil

	N	%	χ^2^	P	Sensitivity	Specificity	Positive predictive value	Negative predictive value
**Total prick test**								
Atopic disease	63	67.0	11.347	0.001	84.0%	47.4%	87.5%	45.4%
Asthma	51	54.3	6.536	0.011	85.0%	38.2%	70.8%	59.1%
Rhinitis	55	58.5	5.611	0.018	83.3%	39.3%	76.4%	50.0%
Atopic eczema	40	42.6	3.798	0.043	85.1%	32.0%	55.5%	68.2%
**Inhaled allergens**								
Atopic disease	27	28.7	9.596	0.002	36.0%	100.0%	100.0%	28.3%
Asthma	22	23.4	5.112	0.024	36.7%	85.3%	81.5%	43.3%
Rhinitis	26	27.7	12.323	< 0.001	39.4%	96.4%	96.3%	40.3%
Eczema	19	20.2	6.287	0.012	83.0%	40.2%	70.4%	58.2%
**Food allergens**								
Atopic disease	59	62.8	7.422	0.006	78.7%	52.6%	86.8%	38.5%
Asthma	49	52.1	5.861	0.015	77.7%	38.7%	72.0%	46.1%
Rhinitis	51	54.3	2.684	0.037	83.6%	48.5%	77.5%	61.5%
Eczema	37	39.4	1.914	0.167	-	-	-	-

A positive SPT was associated with atopic diseases (P = 0.001). Among the patients with positive SPT results, 54.3% reported a history of asthma (P = 0.011), 58.5% reported rhinitis (P = 0.018) and 42.6% reported eczema (P = 0.048) (Table 1).

There was an association between a positive SPT and the following variables: cesarean section (P = 0.015), family history of atopic disease (P < 0.001), maternal asthma (P = 0.022) and pet exposure (P = 0.009) (Table 2).

**Table 2:** Frequency of each factor relating to positive prick tests among 1 to 15-year-old children and adolescents in the city of Palmas, Tocantins, Brazil

Factors	n	%	χ^2^	P
Cesarean delivery	44	46.8	5.826	0.015
Intrauterine tobacco exposure	5	5.3	0.162	0.687
Passive smoking	19	20.2	2.906	0,088
Artificial feeding before six months of life	41	43.6	0.032	0.859
Recurrent infections	20	21.3	1.390	0.859
Familial history of atopic disease	63	67.0	17.170	< 0.001
Premature birth	7	7.4	2.311	0.128
Maternal asthma	20	21.3	5.242	0.022
Gastroesophageal reflux	18	19.1	1.058	0.207
Pet exposure	54	57.4	6.770	0.009

In the multivariate analyses, the dependent variable of allergen sensitization was defined as a positive SPT response. The independent variables that were significantly associated with allergen sensitization in the bivariate analyses were included in the multivariate models. The following factors were significantly correlated with a positive SPT (for food and inhalant allergens): atopic disease (OR = 5.833; CI: 1.958-17.382); asthma (OR = 3.508; CI: 1.303-9.443); rhinitis (OR = 3.235; CI: 1.194-8.769); eczema (OR = 2.679; CI: 1.975-7.357); cesarean birth (OR = 3.367; CI: 1.221-9.288); family history of atopic diseases (OR = 8.400; CI: 2.819-25.029); maternal asthma (OR = 8.077; CI: 1.01864.092); and exposure to pets (OR = 3.600; CI: 1.332-9.731) (Tables 3 and 4).

**Table 3:** Odds ratios for positive skin prick tests and atopic diseases among 1 to 15-year-old children and adolescents in the city of Palmas, Tocantins, Brazil

Atopic disease	Odds ratio	Confidence interval	P
**Total prick test**			
Atopic disease	5.833	1.958-17.382	0.002
Asthma	3.508	1.303-9.443	0.013
Rhinitis	3.235	1.194-8.769	0.021
Atopic eczema	2.679	1.975-7.357	0.015
**Inhaled allergens**			
Atopic disease	-	-	-
Asthma	3.358	1.135-9.934	0.029
Rhinitis	17.550	2.246-137.160	0.006
Atopic Eczema	3.308	1.269-8.624	0.014
**Food allergens**			
Atopic disease	4.097	1.424-11.785	0.009
Asthma	2.238	1.887-5.644	0.008
Rhinitis	2.200	1.849-5.701	0.015
Eczema	1.910	0.759-4.806	0.170

**Table 4:** Odds ratios for positive skin prick tests and risk factors among 1 to 15-year-old children and adolescents in the city of Palmas, Tocantins, Brazil

Factors	Factors	Confidence interval	P
Cesarean delivery	3.367	1.221-9.288	0.019
Intrauterine tobacco exposure	1.567	0.173-14.176	0.689
Passive smoking	3.585	0.765-16.808	0.105
Artificial feeding before six months of life	0.916	0.347-2.414	0.859
Recurrent infections	1.308	0.115-2.235	0.433
Familial history of atopic disease	8.400	2.819-25.029	< 0.001
Premature birth	-	-	-
Maternal asthma	8.077	1.018-64.092	0.048
Gastroesophageal reflux	4.000	0.878-23.181	0.066
Pet exposure	3.600	1.332-9731	0.012

### DISCUSSION

Few studies have assessed sensitization to allergens in primary healthcare units. In our study, there was a significant association between positive skin prick tests and allergic diseases (asthma, rhinitis and atopic eczema). *Dermatophagoides pteronyssinus* was the allergen with the highest prevalence of sensitization among the children who were examined in this study. We observed higher prevalences of asthma, rhinitis and eczema than in the ISAAC study in Brazil,^5^ but our study used an outpatient sample. The northern region of this country has the highest prevalence of asthma and eczema according to the ISAAC study.^5^

The high rates of sensitization and atopic disease may have been due to the characteristics of the outpatient sample. This was recruited in a transitional region between the Cerrado region and the Amazon region during a period of dry weather (September) with a high level of outbreaks of fire. In Palmas, public schools and other buildings are enclosed and air-conditioned, which could have led to increased sensitization to allergens. All of these factors exacerbate respiratory symptoms. Souza et al. reported that there has been an increase in the prevalence and exacerbation of allergic diseases over the last 50 years, especially among children. This increase may have been due to greater exposure to indoor and outdoor pollution, and to lifestyle changes such as increased time spent indoors with air conditioning.^18^

The coexistence of allergic conditions, food allergies, eczema, allergic rhinitis and asthma is increasing.^19^ The present data are largely in agreement with previous reports in the literature, which found that atopic eczema was associated with higher risk of allergic rhinitis and asthma. In a systematic review of worldwide variations in the prevalence of wheezing symptoms in children, Patel et al. observed that the prevalence of childhood asthma has been increasing over time.^20^ The skin prick test is the standard method for allergen sensitization diagnoses, especially with regard to inhalant allergens.^21^

In Singapore, Kidon et al. analyzed 175 patients with an average age of 7.9 years (range 2-16 years). Sixty-eight patients (39%) reported asthma, and 84 patients (48%) reported atopic eczema. Skin prick test results were positive for *Dermatophagoides pteronyssinus* and *Dermatophagoides farinae* in 85% of the patients and *Blomia tropicalis* in 62% of the patients. The prevalence of positive skin prick tests for food allergens was 12%.^22^ Similarly, the average age in our study was 5.34 years (SD ± 3.582), and the prevalence of sensitization to *Dermatophagoides pteronyssinus* was 34%, followed by *Dermatophagoides farinae* at 19.1%, and *Blomia tropicalis* at 18.1%. The prevalence of sensitization to food allergens was 28.9%.

In Malaysia, 141 atopic children were included in a study by Gendeh et al., in which 70% of the atopic children had positive skin prick tests for dust mites and 24.8% of the children tested positive for a shrimp allergy.^1^ Arshad et al. found that allergic disorders were present in 28.1% of 981 children in a cohort study; 19.6% of these patients were atopic and exhibited positive reactions to one or more allergens. Sensitization to inhalant allergens was relatively common (19.2%), compared with food allergens (3.5%).^23^ In our study, cow's milk was the most prevalent food allergen. 

In the Third National Health and Nutritional Examination Survey in the United States, 54.3% of the population had positive test results for one or more allergens, and the prevalences were 27.5% for dust mites, 26.9% for perennial rye, 26.2% for short ragweed, 26.1% for German cockroaches, and 17% for cats.^24^ In Recife, Pernambuco, Brazil, a case-control study found that 27.6% of the children with asthma were positive for *Periplaneta americana* allergies. Sensitization to cockroaches was demonstrated to be a risk factor for greater severity of the disease.^25^ In our study, we found sensitization to *Periplaneta americana* in 8.5% of the patients and German cockroaches in 6.4% of the patients.

Additionally, we found high prevalence of sensitization to grass pollens, which was not expected in a tropical region. This finding may have been due to cross-reactivity between the local outdoor allergens of this region and those of the Amazon Forest and Cerrado. Taketomi et al. reported that patients with grass pollen allergies often present reactivity to pollen allergens from a number of grass species, due to cross-reactivity between IgE antibodies and pollen proteins.^26^ In the state of Rio Grande do Sul in Brazil, Taketomi et al. found that the prevalence of seasonal allergic conjunctivitis was 22.1% among college students in Santo Ângelo and 14.1% in Caxias do Sul.^26^ In a recent study in Curitiba, Paraná, Brazil, Esteves et al. found that the rate of sensitization to *Lolium multiflorum* was 1.8% among teenagers.^27^

We found skin prick test positivity for food allergens among subjects with rhinitis. This result may have been related to the association between rhinitis and eczema (OR = 5.21) and the ages of our patients (a mean age of 5.34 years). Similarly, Kalach et al. conducted a study in Paris and found higher rates of specific IgE positivity for food allergens among children who were four to six years of age, compared with children who were under one year of age.^28^ In Australia, Lowe et al. demonstrated that children with atopic eczema had a greater risk of developing asthma and rhinitis.^29^ Additionally, food allergens can be etiological factors for respiratory and gastrointestinal symptoms.^30^

Paternal atopic disease has been correlated with a higher risk of allergic diseases among children.^31-33^ We found a relationship between skin prick test positivity and a family history of atopic disease and maternal asthma.

Maternal atopic disease has been shown to have significant effects on the patterns of risk factors. Among children with a history of maternal atopic disease, a low birth weight, day care attendance and maternal smoking during the first year of life independently increased the risk of atopic sensitization. Gender, breastfeeding and paternal education did not indicate any association with atopic disease in this group of children, in that study.^34^ It was shown that children of atopic mothers had a different profile of risk factors that were associated with atopic sensitization, compared with other children. Therefore, prenatal and early childhood events were significantly associated with atopic sensitization.^34^

A recent study demonstrated that birth by means of cesarean section was a risk factor for asthma, eczema and hay fever.^35^ Another study reported that cesarean delivery can decrease the production of IFN-g, thereby stimulating the production of Th2 cytokines in the intrauterine environment. The Th2 cytokine environment predisposes individuals towards development of atopy.^36,37^ We observed a relationship between cesarean sections and skin prick test positivity.

However, we did not find any associations between SPT positivity and passive smoking, artificial feeding, precocious egg introduction, intrauterine tobacco exposure or gastroesophageal reflux. On the other hand, several studies have reported associations between SPT positivity and asthma and gastroesophageal reflux.^38^

In study by Ribeiro et al., the presence of respiratory infections and asthma attacks were associated with atopic parents but not with the presence of two or more positive skin tests for allergies among the children who were studied. In addition, the presence of respiratory infections and asthma were not associated with parents who smoked or with studying at boarding school.^33^ However, other studies have found a correlation between asthma and passive exposure to cigarette smoke, especially maternal smoking.^39,40^

Chong Neto et al. found the following risk factors for wheezing within the first year of life: male gender; history of asthma in the family; six or more episodes of cold virus infection; atopic eczema; age at which the child began daycare; and pets in the home during pregnancy.^41^ Ownby et al. reported that exposure to two or more dogs or cats during the first year of life may reduce the subsequent risk of sensitization to multiple allergens during childhood.^42^ Murray et al. reported that exposure to dust mites or cat allergens, but not to dog allergens, was significantly associated with specific allergen sensitization at three years of age. Exposure to dog allergens, but not to dust mites or cat allergens, was significantly associated with wheezing symptoms.^43^ Pet-keeping was associated with doctor-diagnosed asthma (OR = 1.52; 95% CI: 1.25-1.84).^44^ In Brazil, the PROAL study showed that the prevalence of sensitization to cats among atopic children was 12%.^40^ In our study, the presence of pets in the home was a risk factor for atopic diseases, and it is a common habit to keep cats and dogs in homes in Palmas, Tocantins, Brazil. 

Kull et al. reported that breast-feeding reduces the risk of childhood eczema and delayed the onset of allergies.^45^ In a study by Snijders et al., longer duration of breastfeeding decreased the risk of recurrent wheezing, which was independent of maternal allergies. Their findings suggested that the relationship between breastfeeding and infant eczema during the first two years of life was modified by the maternal allergic status. The protective effect of breastfeeding on recurrent wheezing may be associated with protection against respiratory infections.^46^ In our study, there was no correlation between breast-feeding and a positive skin prick test.

Finally, Souza et al. emphasized the following findings regarding allergen sensitization: "knowledge of allergen exposure in different environments is essential to identify clinically relevant allergens and then supply adequate treatment to allergic patients. Because this is a dynamic process with many variables that change over the years, periodic assessment of environmental allergens is necessary to establish changes in the profile and dispersion of allergens in each region."^18^

### CONCLUSION

In summary, dust mites (*Dermatophagoides pteronyssinus*) were the most frequent allergen and cow's milk was the most prevalent food allergen among children who were 1-15 years of age at two outpatient clinics in Palmas, Tocantins, Brazil. There was a relationship between a positive skin prick test and the prevalence of allergic diseases (asthma, rhinitis and eczema) and other factors, such as a family history of atopic disease, maternal asthma, pet exposure and cesarean delivery. The association observed is in agreement with previous studies but is unprecedented for the state of Tocantins (which is in a region of transition between the Cerrado and the rainforest, i.e. the Amazon Forest). The observed risk factors could be used to screen children with a higher risk of allergic diseases, in order to promote early diagnosis.

Overall, the results provide evidence of the pattern of SPT sensitization among children who are examined at primary care centers. From this study, the findings of sensitization to the observed allergens and the factors that were associated with atopy among the children at these outpatient clinics will contribute towards development of preventive actions in the state of Tocantins. Future research should assess the profile of allergen sensitization among atopic children in this community. In addition, these studies should determine the impact of preventive measures against such allergens and control over the factors that are associated with development of atopic diseases.
